# Signal Peptide-Dependent Inhibition of MHC Class I Heavy Chain Translation by Rhesus Cytomegalovirus

**DOI:** 10.1371/journal.ppat.1000150

**Published:** 2008-10-03

**Authors:** Colin J. Powers, Klaus Früh

**Affiliations:** Oregon Health and Science University, Vaccine and Gene Therapy Institute, Beaverton, Oregon, United States of America; University of Southern California School of Medicine, United States of America

## Abstract

The *US2-11* region of human and rhesus cytomegalovirus encodes a conserved family of glycoproteins that inhibit MHC-I assembly with viral peptides, thus preventing cytotoxic T cell recognition. Since HCMV lacking *US2-11* is no longer able to block assembly and transport of MHC-I, we examined whether this is also observed for RhCMV lacking the corresponding region. Unexpectedly, recombinant RhCMV lacking *US2-11* was still able to inhibit MHC-I expression in infected fibroblasts, suggesting the presence of an additional MHC-I evasion mechanism. Progressive deletion analysis of RhCMV-specific genomic regions revealed that MHC-I expression is fully restored upon additional deletion of *rh178*. The protein encoded by this RhCMV-specific open reading frame is anchored in the endoplasmic reticulum membrane. In the presence of rh178, RhCMV prevented MHC-I heavy chain (HC) expression, but did not inhibit mRNA transcription or association of HC mRNA with translating ribosomes. Proteasome inhibitors stabilized a HC degradation intermediate in the absence of rh178, but not in its presence, suggesting that rh178 prevents completion of HC translation. This interference was signal sequence-dependent since replacing the signal peptide with that of CD4 or murine HC rendered human HCs resistant to rh178. We have identified an inhibitor of antigen presentation encoded by rhesus cytomegalovirus unique in both its lack of homology to any other known protein and in its mechanism of action. By preventing signal sequence-dependent HC translocation, rh178 acts prior to US2, US3 and US11 which attack MHC-I proteins after protein synthesis is completed. Rh178 is the first viral protein known to interfere at this step of the MHC-I pathway, thus taking advantage of the conserved nature of HC leader peptides, and represents a new mechanism of translational interference.

## Author Summary

To avoid immune detection by cytotoxic T lymphocytes, viruses interfere with antigen presentation by major histocompatibility complex class I (MHC-I) molecules. We have discovered a unique cytomegaloviral protein that interferes with the biosynthesis of MHC-I heavy chains and was thus termed viral inhibitor of heavy chain expression (VIHCE). We show that VIHCE does not affect transcription of MHC-I mRNA or the formation of poly-ribosomes. Surprisingly, however, very little MHC-I protein is detected, even when proteasomal protein degradation is inhibited, suggesting incomplete protein translation. Interestingly, VIHCE requires the proper MHC-I signal peptide, suggesting that CMV takes advantage of the high conservation of MHC-I signal peptides and interferes with protein translation by inhibiting signal sequence-dependent protein translocation. This is the first description of a viral protein that specifically targets the translation of a cellular immuno-stimulatory protein.

## Introduction

Human cytomegalovirus (HCMV) is a widespread pathogen which is mostly asymptomatic in immune competent individuals, but pathogenic in the immune compromised such as post-transplant or AIDS patients [1]. Following primary infection, HCMV establishes a latent infection for life which is largely controlled by the cellular immune system. Immune control of HCMV requires enormous immunological resources with often more than 10% of the T cell pool being CMV-specific, a number that might further increase with age [2]. However, these immunological efforts are unable to eliminate the virus and do not prevent super-infection [3]. Thus, HCMV is a master in surviving in the face of a constant immunological onslaught.

As one of the largest human viruses, with well over 200 open reading frames (ORFs), HCMV uses only about a third of its coding potential for “essential” functions whereas the majority of its genes are non-essential for growth *in vitro* [4],[5]. Many of these “non-essential” genes encode modulators of innate or adaptive immune responses including inhibitors of apoptosis, interferon-induction, T cell and NK cell recognition [6]–[9]. However, the importance of these immune modulators for viral pathogenesis and immune escape *in vivo* is not known since HCMV does not infect immunocompetent experimental animals. Such restricted species specificity is a hallmark of CMVs and, as a result, CMVs have co-evolved with their hosts [10]. Chimpanzee CMV is most closely related to HCMV [11]. However, chimpanzees are a protected species and unsuitable as an animal model. Although more distantly related to humans, rhesus macaques (RM) are readily available for experimentation. Sequence analysis of rhesus CMV (RhCMV) revealed that approximately 60% of the open reading frames (ORFs) are homologous to HCMV ORFs including most of the aforementioned immune modulators [12],[13]. In order to study the importance of some of the immune regulatory functions *in vivo*, we have begun to characterize several of the conserved immune modulators of RhCMV.

The US2-US11 genomic region of HCMV encodes multiple proteins that interfere with several MHC and MHC-like molecules. Among the best studied of these is the US6-family which contains four genes that inhibit MHC class I (MHC-I)-mediated antigen presentation to T cells: US2, US3, US6 and US11 [14]–[16]. These proteins are type I transmembrane glycoproteins that reside in the endoplasmic reticulum and show clear homology to each other and structural features resembling the IG-superfamily fold [17]. Despite these structural similarities, each protein interferes in its own unique way with the assembly of MHC-I with peptides at a post-translational level. Upon completion of heavy chain (HC) translation and translocation into the lumen of the ER, but prior to assembly with the light chain β2-microglobulin (β2-m), US2 and US11 mediate the retro-translocation of MHC-I molecules to the cytosol [18]. There, the HC is deglycosylated by N-glycanase and degraded by the proteasome [19]. US6 inhibits peptide translocation by the TAP thus preventing the MHC-I heterodimers from obtaining viral peptides [16]. Finally, US3 prevents ER exit of peptide-loaded MHC-I molecules [15], both by directly interacting with MHC-I molecules and by interfering with tapasin and protein-disulfide isomerase, both chaperones of the peptide loading complex [20].

We previously demonstrated that the US2-11 orthologues of RhCMV are also functionally equivalent in that Rh182 (US2) and Rh189 (US11) mediate proteasomal destruction of MHC-I, Rh183 (US3) retains MHC-I and Rh185 (US6) inhibits TAP [21]. Thus, it seemed likely that eliminating the genomic region spanning *RhUS2-11* from RhCMV would restore MHC-I assembly and transport in RhCMV-infected cells as previously observed for *US2-11*-deleted HCMV [22]. Surprisingly however, we discovered that in addition to these conserved mechanisms, RhCMV contains an additional ORF, *rh178*, that targets the MHC-I assembly pathway. Interestingly, this ORF does not display any homology to the US6 gene family and acts by a novel mechanism that operates post-transcriptionally, but prior to completion of translation/translocation.

## Results

### Inhibition of MHC-I expression despite deletion of RhUS2-US11

**Figure 1:**
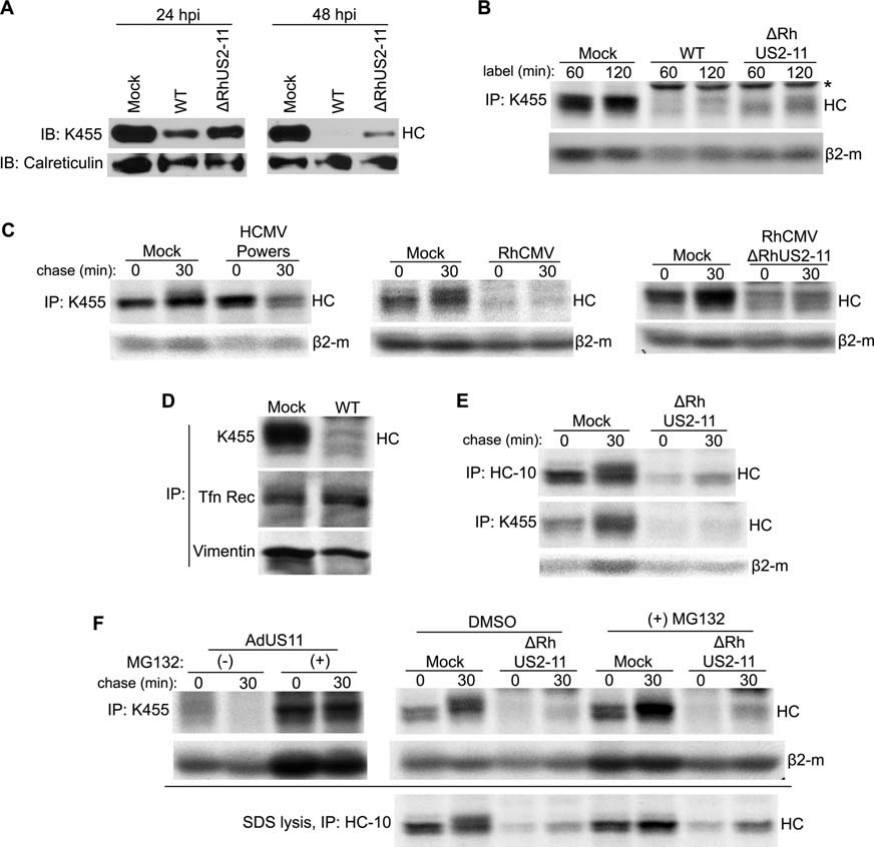
RhCMV inhibits HC expression in the absence of RhUS2-11. All experiments were performed at 24 hours post infection at MOI = 3. A) Immunoblot analysis of MHC-I or calreticulin in Mock- or RhCMV-infected TRF lysates. B) IP of total MHC-I upon labeling with ^35^S-Met/Cys for the indicated time. (*) All IPs from WT and recombinant RhCMV- infected cells contain antibody-binding proteins around 55kDa (see Fig. S2) which likely correspond to the RhCMV homologues of the Fc-receptor UL119-118 of HCMV [50]. Since these viral proteins are not involved in MHC-I inhibition they are not shown in most figures. C) Pulse-chase labeling of 10 min and immunoprecipitation of total MHC-I from Mock-infected, HCMV-infected THFs, or RhCMV-infected TRFs. D) Pulse-labeling of 60 min and IP of MHC-I, Tfn Rec (Transferrin receptor) or Vimentin from Mock-infected or RhCMV-infected TRFs. E) Pulse-chase labeling of 10 min and IP of total MHC-I or HC. Cells were labeled as in 1C, but lysed in SDS buffer prior to IP. F) Pulse-chase labeling and IP of RhCMV-infected TRFs treated with proteasome inhibitor. Where indicated TRFs were incubated with 50 µM MG132 or DMSO during 60-min of Met/Cys starvation, 10-min label, and 30-min chase. For control, TRFs were transduced with AdUS11 (MOI = 25), a recombinant adenovirus expressing HCMV US11, for 24 hours followed by NP40-lysis and IP with K455. Shown for RhCMV-infection is both NP-40 lysis (top panel) and SDS-lysis (bottom panel) prior to IP with the noted antibody.

Deletion of the genomic region encoding US2-US11 restores MHC-I expression in HCMV-infected cells [22]. To determine if deletion of the homologous region in the RhCMV genome would likewise restore MHC-I expression we created a recombinant RhCMV lacking RhUS2-11 using a RhCMV-derived bacterial artificial chromosome (BAC) (Protocol S1) [23]. Similar to recombinant HCMV lacking US2-11, a growth defect was not observed for ΔRhUS2-11 [22]. However, unlike US2-11-deleted HCMV, ΔRhUS2-11 retained some ability to reduce MHC-I steady state levels in infected TRFs as shown by immunoblot (Fig. 1A). At 48 hours post-infection, MHC-I was markedly reduced in ΔRhUS2-11-infected TRFs.

To determine whether the reduced steady state levels were due to interference with newly synthesized MHC-I, we immunoprecipitated MHC-I from radiolabeled TRFs infected with wild-type (WT) or ΔRhUS2-11. When cells were labeled for one or two hours, we recovered dramatically less MHC-I from RhCMV-infected cells despite the use of polyclonal antiserum K455 recognizing all forms of MHC-I (Fig. 1B) [24]. Compared to WT there was an increase in HC recovery from ΔRhUS2-11-infected cells. Such residual HC was also observed in pulse-chase experiments, when ΔRhUS2-11-infected TRFs were pulsed for 10 min and chased from 30 min up to 90 min (Fig. S1A). However, compared to mock-treated cells, radiolabeled HC was drastically reduced at all time points either during pulse or chase. In contrast to HC, expression of control proteins such as Transferrin-receptor or vimentin was unaffected in RhCMV-infected cells (Fig. 1D). Also, we did not observe a general shut-off of host protein expression or a dramatic decrease of glycoprotein recovered with the lectin concanavalin A (data not shown). Moreover, expression of the light chain β2-m was much less affected by RhCMV compared to HC, particularly in short pulse/chase experiments (Fig. 1C). These data suggested that in addition to RhUS2-11 inhibiting MHC-I assembly, RhCMV specifically interferes with expression of HC. The residual HC recovered from ΔRhUS2-11-infected cells indicate that this viral inhibition of HC expression (VIHCE) was either incomplete or VIHCE did not equally affect all MHC-I alleles present in TRFs.

### VIHCE does not cause rapid degradation of complete HCs

Since only minimal amounts of HC are detectable during ΔRhUS2-11 infection, we wanted to examine if VIHCE caused rapid degradation of HCs. In cells infected with HCMV, HC is initially synthesized but then rapidly degraded as shown by pulse-chase (Fig. 1C). This observation is consistent with previous reports and is due to the reverse translocation of MHC-I mediated by US2 and US11 followed by proteasomal destruction of MHC-I [19]. In contrast, during infection with both WT RhCMV and ΔRhUS2-11 only minimal amounts of HC were detectable after a 10-min radiolabel, and remained low during a 30-min chase (Fig. 1C). Furthermore, during a radiolabel for only 1-min HC synthesis was markedly reduced during RhCMV infection (Fig. S1B). To rule out that HC was not recovered due to epitope masking by a viral protein or because HC was in a complex with NP40-insoluble proteins, we lysed cells in SDS to disrupt protein complexes and denature the HC prior to IP. Using either a monoclonal antibody that recognizes only free HC (HC-10) [25] or K455, we were unable to recover increased amounts of HC under these conditions (Fig. 1E). Taken together these data suggest that RhCMV either prevents complete HC synthesis or degrades HC prior to complete protein synthesis.

Since co-translational degradation is mediated by proteasomes [26] we wanted to determine whether HC translation could be completed in the presence of proteasome inhibitors. TRFs were infected with ΔRhUS2-11 and treated with the proteasomal inhibitor MG132. However, no significant increase in HC recovery was observed either when total MHC-I was immunoprecipitated with K455 from NP40-lysates or with HC-10 from SDS-lysates (Fig. 1F). In contrast, HC was stabilized in cells transduced with Adenovirus expressing HCMV US11. The proteasomal inhibitors Lactacystin and ZL_3_VS also failed to stabilize HC in ΔRhUS2-11-infected cells (data not shown). Taken together these data strongly suggest that RhCMV inhibits expression of HC prior to or during polypeptide synthesis. Since this phenotype is observed in the absence of RhUS2-11 and is not present in HCMV, we further conclude that RhCMV contains one or more unique gene(s) encoding VIHCE.

### HC synthesis is restored upon infection with RhCMV lacking *Rh158–180*

**Figure 2:**
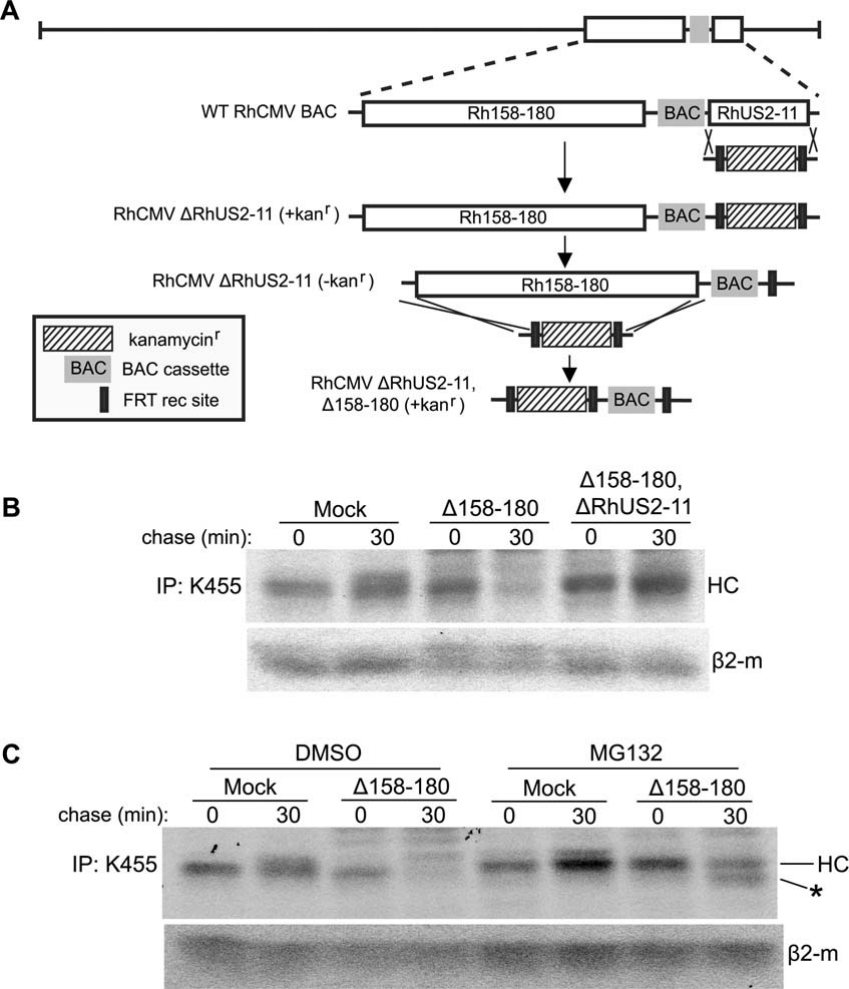
Deletion of Rh158–180 restores MHC-I expression during RhCMV infection. A) Diagram of the step-wise construction of the ΔRhUS2-11 and Δ158–180,ΔRhUS2-11 viruses. Using the RhCMV BAC the RhUS2-11 region was replaced with a PCR-fragment containing a Kanamycin resistance (Kan^r^) cassette flanked by RhCMV homologous regions. The Kan^r^ cassette was removed by arabinose-induced FLP recombinase prior to replacing the Rh158-180 region with Kan^r^. B) Pulse-chase labeling for 10 min of TRFs infected with WT or recombinant RhCMV followed by IP of total MHC-I. In C) 50 µM MG132 or DMSO was included as in Fig. 1F. (*) indicates a deglycosylated cytosolic degradation intermediate stabilized by MG132.

Since VIHCE seems to be specific to RhCMV, but absent from HCMV, we examined the RhCMV genome for potential candidate genes. The genomic region spanning ORFs *Rh158* to *rh180*, corresponding to the region between *IE1/IE2 (UL123/UL122)* and *US1* in HCMV, contains a large number of genes that are either specific to RhCMV or are homologous to genes frequently deleted in laboratory strains of HCMV [12],[27]. To examine whether this region contains the VIHCE gene, we deleted *Rh158–180* using the BAC-recombination strategy shown in Fig. 2A. Interestingly, Δ158–180 did not show any obvious growth defects despite such a large deletion (data not shown). Moreover, pulse-chase labeling of Δ158–180-infected TRFs revealed initial synthesis of MHC-I followed by degradation (Fig. 2B). This degradation could be inhibited by the proteasome inhibitor MG132 (Fig. 2C). MG132 also stabilized a smaller, presumably deglycosylated, degradation intermediate (*) which is also observed in cells transfected with RhUS2 [21]. Thus, it seemed likely that Δ158–180 lacked VIHCE, and that in the absence of VIHCE HC was now degraded by the RhCMV homologues of US2 and US11. To examine whether the combined deletion of *RhUS2-11* and VIHCE would restore HC expression in RhCMV-infected cells, we created a recombinant lacking both *Rh158–180* and *RhUS2-11* (Fig. 2A). As expected from the single deletions, the resulting double-deletion virus Δ158–180,ΔRhUS2-11 did not display a growth defect *in vitro* (not shown). When TRFs were infected with Δ158–180,ΔRhUS2-11, HC expression was similar to Mock-infected cells indicating that this recombinant virus no longer interfered with MHC-I expresson (Fig. 2B). Taken together, these data indicate that the VIHCE gene is located within the *Rh158–180* region of RhCMV. Furthermore, the fact that HC synthesis is observed in the absence of VIHCE supports our conclusion that VIHCE acts prior to the ER-associated degradation caused by the US2-US11 homologs.

### RhCMV VIHCE maps to *rh178*

**Figure 3:**
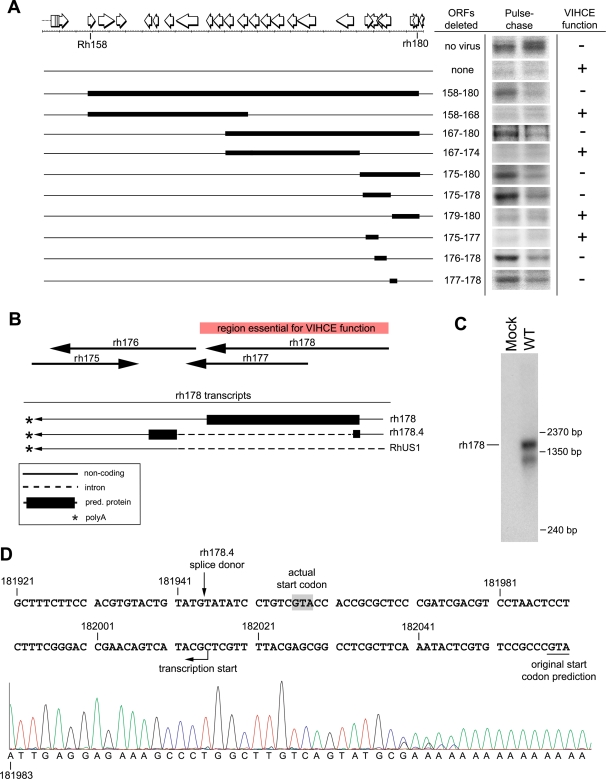
VIHCE is encoded by *rh178*. A) Deletional mapping of VIHCE. Predicted open reading frames between Rh158–180 are shown as open white arrows. Solid black rectangles indicate the region of deletion. Pulse-chase labeling for 10 min with the indicated recombinant virus was performed as in Fig. 1C followed by IP with K455. Lack of VIHCE is readily apparent by the initial synthesis of HC (left lane) followed by US2-11-mediated destruction (right lane). B) Predicted ORFs and experimentally confirmed transcripts in the *rh178* region. The red rectangle indicates the region essential for VIHCE function as determined by deletions in several independent recombinants. Large black arrows indicate positions of ORFs *rh175–178* predicted by [12]. Transcripts confirmed by RACE and cDNA PCR are shown below. C) Northern blot analysis of total RNA isolated from mock or WT RhCMV-infected TRFs at 24 hours post infection. ORF *rh178* was used to generate ^32^P-dCTP labeled DNA probe. D) Complementary sequence of the RhCMV genome from 181921–182060bp. Underlined at 182058bp is the original predicted start codon for rh178 [12]. Transcription actually begins at 182015bp as determined by 5′ RACE (see sequence chromatogram below genomic sequence). Shaded in gray is the first ATG codon of the transcript. Also noted is the splice donor site for *rh178.4* which is spliced at 181944bp.

To identify the gene(s) coding for VIHCE we systematically deleted fragments of decreasing size within the *Rh158–180* region in an iterative fashion (Fig. 3A; Table S1). We took advantage of the fact that HC is initially synthesized in cells infected with VIHCE-deleted virus but then degraded by US2 and US11 to distinguish between recombinants encoding or lacking VIHCE. Initially, viruses carrying deletions approximately spanning the left or right half of the *Rh158–180* region were generated (Fig. 3A). TRFs were infected with recombinants Δ158–168 and Δ167–180 and pulse-chase was performed. Since HC was expressed in TRFs infected with Δ167–180 and not in TRFs infected with Δ158–168, we concluded that VIHCE was located in the *Rh167–180* region. Similarly, HC was expressed in TRF infected with viruses Δ175–180, Δ175–178, Δ176–178, and Δ177–178, but not Δ167–174, Δ179–180, and Δ175–177 (Fig. 3A). These data suggested that *rh178* encodes VIHCE.

**Figure 4:**
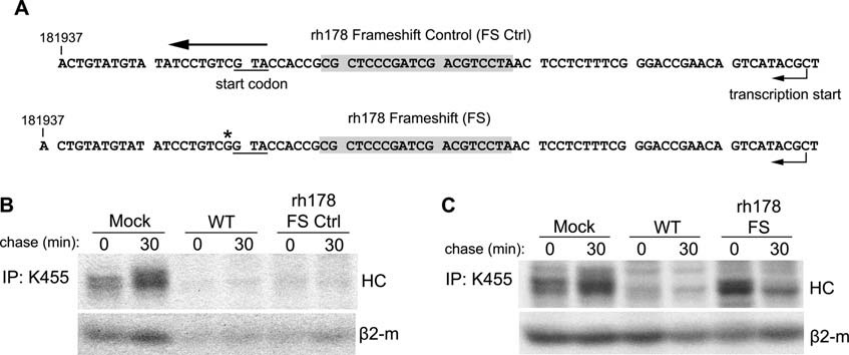
A frameshift mutation of *rh178* restores HC expression. A) Sequence of the rh178 frameshift control and frameshift recombinants. Shown is complementary genomic sequence, with transcripts running from right to left. In each recombinant a 20bp sequence in the 5′ UTR of *rh178* (gray boxes) was replaced with 93bp from the recombination vector including the FRT recombination site. (*) indicates the single base insertion causing a frameshift. B) and C) HC expression in TRFs infected with control or frameshift viruses. Pulse-chase and IP was performed as in Fig. 1C.

The region encoding *rh178* overlaps with several predicted ORFs and with a previously identified large intron of the *US1*-homologue *Rh181* [28] (Gene Bank Accession: AF474179). To exactly determine the mRNAs encoding VIHCE we mapped the transcriptional start and stop sites of the *rh178* ORF and generated additional, smaller deletions and point mutants within the *rh178* coding region (Figs. 3–4). We performed 5′ and 3′ RACE as well as Northern blot analysis. Sequence analysis of the 5′ RACE product identified a transcription start site downstream of the originally predicted *rh178* start codon (Fig. 3D). The identified transcript is predicted to encode a shorter version of rh178. 3′RACE and cDNA cloning further revealed additional splice products in this region: a shorter splice product lacking most of the *rh178* protein encoding region (*rh178.4*; Note that Rivailler et al., (2006) have detailed additional predicted ORFs upstream of *rh178* and denoted them *rh178.1, rh178.2*, and *rh178.3*) and the above mentioned large *Rh181*-transcript which does not contain *rh178* since it is removed by splicing. These three transcripts share the same polyadenylation signal and 3′ terminus (Fig. 3B). Northern blot analysis using the predicted *rh178* coding region as probe revealed two transcripts (Fig. 3C). A larger predominant transcript of approximately 1600bp corresponds to the expected size of *rh178*. The smaller transcript may correspond to *rh178.4*, a shortened *rh178*, or an unidentified transcript of the opposite sense. These data confirm the expression of the *rh178* transcript during infection and correct the prediction of its protein coding region.

Kinetic analysis indicates that VIHCE is an early gene that is already expressed within 4 hours of infection (Fig. S3). To determine whether the protein encoded by *rh178* is responsible for VIHCE, we created a frameshift in the 5′-end of the predicted coding region (rh178FS) (Fig. 4A). Since the primer-directed mutagenesis strategy also caused deletion of a portion of the 5′-UTR we generated a control virus (rh178FSCtrl) containing the same modification of the predicted 5′-UTR of *rh178* but no frameshift (Fig. 4A). While rh178FSCtrl inhibited HC expression similar to WT (Fig. 4B), HC was synthesized in rh178FS-infected TRFs (Fig. 4C). Thus, VIHCE is mediated by the *rh178*-encoded protein.

**Figure 5:**
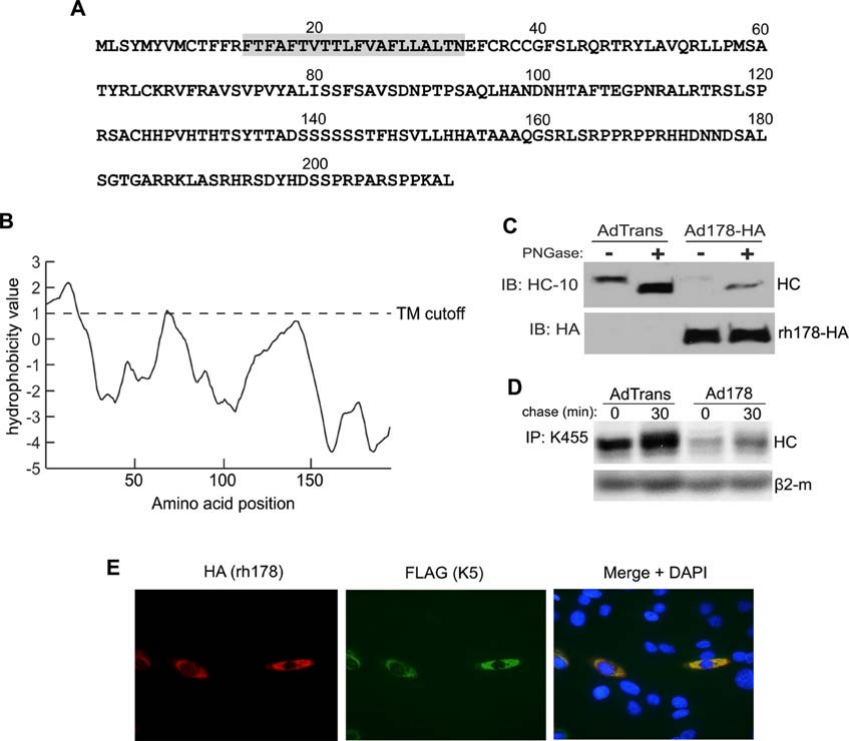
rh178 is a 212aa non-glycosylated ER localized protein that is sufficient to block HC synthesis. A) Complete polypeptide sequence of rh178. Shaded in gray is the predicted signal anchor sequence. B) Hydrophobicity graph of rh178 (TopPred, http://bioweb2.pasteur.fr/). TM refers to a predicted transmembrane domain cutoff value. C) Western blot of lysate from TRFs transduced with replication deficient adenovirus vectors AdTrans (expressing a tetracycline responsive transactivator) or AdTrans together with Ad178-HA (expressing HA-tagged rh178) for 48 hours. Lysate was treated without or with PNGase to remove N-linked sugars and blotted for MHC-I HC using the HC-10 antibody or for rh178-HA with an anti-HA antibody. D) HC expression in TRFs transduced with AdTrans or AdTrans with Ad178 (expressing wild type rh178) for 24 hours, followed by a 10-min pulse label and 30-min chase. HCs were recovered with K455 from NP40 lysates. E) Immunofluorescence analysis of TRFs 24 hours after transfection with HA-tagged rh178 together with FLAG-tagged K5 from KSHV. Primary antibodies were mouse anti-FLAG and rabbit anti-HA. Secondary antibodies were 594 Alexa Fluor conjugated goat anti-rabbit and 488 Alexa Fluor conjugated goat anti-mouse.

The rh178 protein (Fig. 5A), with a molecular weight of approximately 24 kDa, does not display significant homology with non-RhCMV sequences in the genomic database. A stretch of highly hydrophobic amino-acids beginning at amino acid 14 is predicted to represent a non-cleaved amino-terminal signal anchor (Fig 5B). Thus, the most likely topology for this protein is that of a type 1b transmembrane protein, i.e. a large cytoplasmic C-terminus following the signal-anchor. Immunofluorescence analysis of epitope-tagged rh178 indicates that the protein localizes to the ER, suggesting that rh178 is anchored in the ER-membrane (Fig. 5E). To obtain better expression of rh178 for further analysis, we constructed replication-defective adenovirus vectors expressing either wild type rh178 (Ad178) or HA-tagged rh178 (Ad178-HA). While there is a predicted glycosylation site at position N101, digestion of whole cell lysate from Ad178-HA transduced cells with peptide:N-Glycosidase F (PNGase) failed to cause a shift in rh178 migration, while a shift was seen with MHC-I HC (Fig. 5C). Thus, rh178 does not appear to be glcosylated and this is a further indication that the C-terminus of rh178 is located in the cytosol. To determine if rh178 by itself was capable of VIHCE, we transduced TRFs and performed pulse-chase analysis. Cells transduced with Ad178 exhibited reduced expression of HCs while β2-m was unaffected (Fig. 5D), similar to the HC inhibition observed in RhCMV-infected cells (Fig. 1). MHC-I HC in cells transduced with a control adenovirus vector, AdTrans, was not affected. Thus, rh178 is both necessary and sufficient for VIHCE.

### RhCMV does not inhibit transcription or ribosome association with HC mRNA

**Figure 6:**
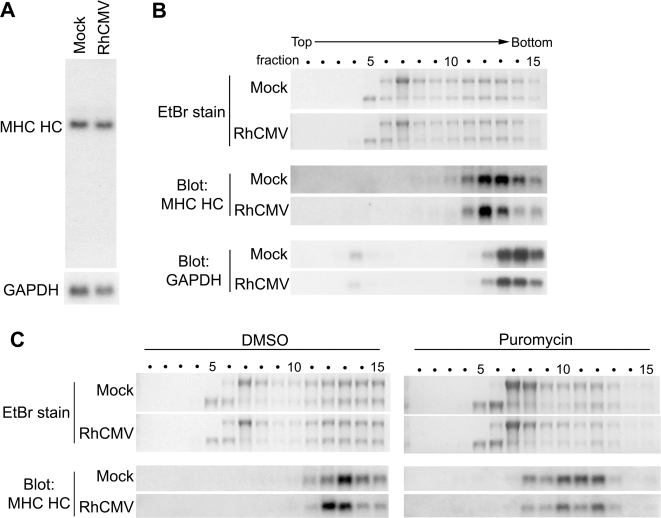
HC mRNA is present, intact, and associates with actively translating ribosomes during RhCMV infection. A) Northern blot analysis of HC- or GAPDH-specific mRNA from total RNA isolated at 24 hours after Mock- or RhCMV-infection. The ^32^P-dCTP labeled probes were generated using rhesus-derived cDNAs for HC or GAPDH as templates. B) Polyribosome fractionation and northern blot analysis. TRFs were either mock infected or infected with wild-type (WT) RhCMV at MOI = 3 for 24 hours followed by isolation and fractionation of polysomes. Ethidium Bromide (EtBr) staining of a denaturing agarose gel shows the amount and ratio of 18S and 28S rRNA present in each fraction, indicating the presence of ribosomal subunits. Polysomes sediment to higher, denser fractions. Lower panels show northern blots of the gel using the HC and GAPDH-specific probes. C) Cells were infected as in B. However, after 24 hours, cells were incubated for 4 min with either DMSO or 100 µg/ml puromycin prior to polysome harvesting.

Our data suggest that VIHCE prevents expression of the majority of HCs prior to completion of protein synthesis. Residual, VIHCE-resistant HCs are eliminated by RhUS2-11. The dramatic reduction of newly synthesized HC observed even in the presence of proteasome inhibitors further suggests that VIHCE either blocks transcription of HC mRNA, completion of HC protein synthesis, or causes HC degradation in a proteasome-independent manner. However, the levels of HC mRNA did not change upon RhCMV-infection as shown by Northern blot (Fig. 6A) and by quantitative RT-PCR (data not shown). Additionally, the size of the HC mRNA was unaltered in RhCMV-infected cells suggesting that mRNA is not cleaved, alternative spliced or degraded by RhCMV. We further determined whether HC mRNA is polyadenylated and exported into the cytoplasm by isolating nuclear, cytoplasmic, and polyadenylated RNA fractions from infected cells. We did not observe a significant difference in any of these fractions compared to Mock-infected cells (data not shown). These data indicate that HC mRNA transcription, poly-adenylation, splicing and export to the cytosol is not affected by RhCMV.

To determine whether the association of HC mRNA with ribosomes is inhibited we analyzed the polyribosome distribution of HC mRNA [29]. When sucrose-gradient fractions from lysates of Mock-infected or RhCMV-infected TRFs were analyzed by Northen blots, HC mRNA sedimented to the polyribosome fractions 12 and 13 in both Mock- and RhCMV-infected cells (Fig. 6B). Small shifts in polyribosome density were observed in RhCMV infection for both HC and GAPDH mRNA, suggesting virus infection causes a slight reduction of ribosomal occupancy on cellular transcripts. Therefore, it seems that VIHCE does not inhibit the association of polyribosomes with HC mRNA.

While sedimentation to the polyribosome fraction indicates the association of HC mRNA with ribosomes, it was possible that the ribosomes were not active. In order to determine if the ribosomes associated with the HC mRNA are actively translating we incubated cells with puromycin. Puromycin is a polypeptide chain terminator that requires an active peptidyl transferase to cause ribosome dissociation from transcripts. A short (4 min) incubation with puromycin caused a shift in the polyribosome profile of HC mRNA in both RhCMV and Mock-infected cells, indicating ribosome dissociation (Fig. 6C). This result indicates that the ribosomes bound to the HC mRNA are actively translating and not simply stalled on the transcript. Taken together these data suggested that HC mRNA is transcribed normally in RhCMV-infected cells and that protein translation is not inhibited at the level of initiation or elongation. However, since full-length HC protein cannot be recovered it seems most likely that HC translation is not completed.

### VIHCE is dependent upon the MHC-I signal peptide

**Figure 7:**
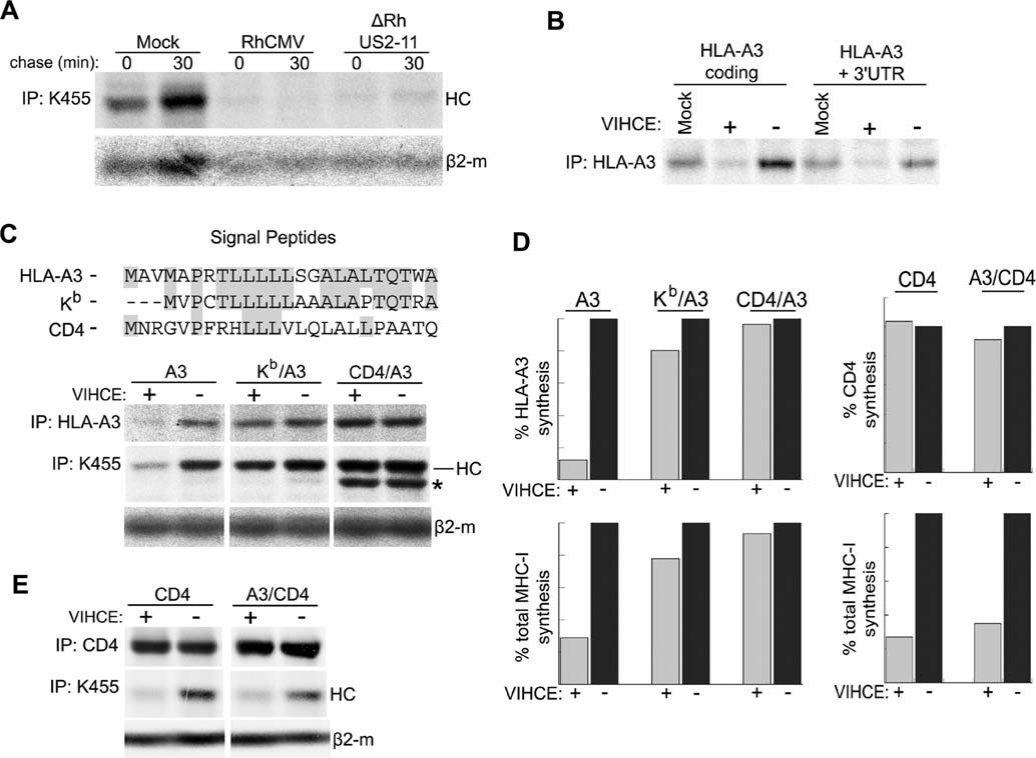
Efficient HC targeting by rh178 is signal-peptide dependent. A) Rh178 inhibits expression of human HC. THFs were infected with the indicated virus at MOI = 3 for 24 hours, followed by a 10-min pulse-label, a chase of 30-min and IP with K455. B) UTR-independent inhibition of HLA-A3 expression by rh178. TRFs were electroporated with pEF1α containg the indicated HLA-A3 construct. After 24 hours, cells were either mock infected or infected with recombinant RhCMV (MOI = 3) containing VIHCE (+; ΔRhUS2-11) or lacking VIHCE (−; Δ178, ΔRhUS2-11). After an additional 24 hours, cells were labeled for 30-min, lysed in NP-40, and HLA-A3 was immunoprecipitated. C) Upper panel: Amino acid sequence of the signal peptides used in chimeric HLA-A3 HCs. Gray shading indicates identity with the HLA-A3 signal peptide. Lower panel: TRFs were electroporated with native HLA-A3 (A3) or the indicated SP-chimera (the HLA-A3 signal peptide was replaced with the H2-K^b^ or the CD4 signal peptide in K^b^/A3 or CD4/A3, respectively) prior to infection with RhCMV, metabolic labeling and IP as in 7B. (*) indicates an uncharacterized HC-band that appears prominently in IPs from CD4/A3 transfectants and that could represent a deglycosylated or truncated HC. D) Quantitation of HLA-A3, total HC, or CD4 expression from 7C and 7E shown as a percent relative to HC or CD4 levels in the absence of VIHCE. Bands were quantitated using ImageQuant 5.1 software (Molecular Dynamics). E) TRFs were electroporated with native CD4 or CD4 containing the HLA-A3 SP (A3/CD4) and treated as in 7C. All experiments are representative of several replicates.

Observations similar to VIHCE were reported for translation inhibition by microRNAs that bind to the 3′-UTR of target transcripts. Similar to VIHCE, mRNAs that are targeted by a given microRNA are found in an active polyribosomal complex but a translated polypeptide intermediate can not be recovered even in the presence of proteasome inhibitors [30]. To examine the possibility that VIHCE targets the 3′-UTR of HCs we tested the ability of VIHCE to block synthesis of HC with or without its native 3′-UTR. Since antibodies to rhesus HCs are not available, and VIHCE is able to block expression of human HCs (Fig. 7A), we chose to examine VIHCE function on HLA-A3. To determine whether the 3′-UTR was required for this inhibition we transiently expressed HLA-A3 with or without its native 3′-UTR in TRFs. Following transfection we infected cells with either RhCMV containing VIHCE (ΔRhUS2-11) or RhCMV lacking VIHCE (Δrh178,ΔRhUS2-11). Expression of both HLA-A3 carrying the native 3′-UTR and a heterologous vector-derived 3′-UTR sequence was reduced by VIHCE (Fig. 7B). The 5′-UTR was vector-derived in both constructs. Therefore, we conclude that VIHCE does not target the UTRs of HC mRNA.

Translation of type I transmembrane proteins such as HC is dependent upon an N-terminal signal peptide (SP) that mediates translocation across the ER membrane. Upon translation initiation, the SP is recognized by the signal-recognition particle (SRP) which binds to the SP and arrests translation. This is followed by docking of the translation complex to the SRP-receptor which aids the transfer of the ribosomal/mRNA/nascent polypeptide complex to the SEC61 translocon [31]. Translation then resumes and the nascent polypeptide chain is imported into the lumen of the ER. The fact that VIHCE requires the HC coding sequence suggested that the HC protein might be at least partially translated and that VIHCE acts on the nascent polypeptide. Compared to human HC, we observed that the murine MHC-I molecule H2-K^b^ was more resistant to VIHCE (data not shown). We hypothesized that this resistance was encoded in the amino-terminus of H2-K^b^, specifically the SP. To test this hypothesis we replaced the SP of HLA-A3 with that of H2-K^b^. As a further control, we also introduced the SP of CD4 which is more divergent from the HLA-A3 SP (Fig. 7C). In both instances we observed that expression of the chimeric protein was much less reduced by virus expressing VIHCE compared to native HLA-A3. Remarkably, the SP of Kb is quite similar to that of HLA-A3 (Fig. 7C) yet HLA-A3 expression was restored to almost the same levels as observed for the CD4 SP (Fig. 7D). Therefore, we conclude that the SP of primate MHC-I is required for VIHCE to inhibit HC translation. The fact that VIHCE requires the MHC-I SP further suggests that VIHCE interferes with SP-dependent translocation which would lead to translation arrest and rapid, co-translational destruction of the resulting protein fragments.

We next examined if the MHC-I SP is sufficient for VIHCE recognition. To test this we created a chimeric CD4 molecule with the HLA-A3 signal peptide in place of the native CD4 signal peptide (A3/CD4). When either wild type CD4 or A3/CD4 was expressed in TRFs, neither molecule was significantly affected by the presence of VIHCE, whereas the endogenous MHC-I HC was decreased (Fig. 7E, 7D). This indicates that while the MHC-I SP is necessary for recognition by VIHCE, it is not entirely sufficient.

## Discussion

We report here that the ORF *rh178* of RhCMV encodes a novel immune modulatory function, viral inhibitor of heavy chain expression (VIHCE), which prevents the translation of HC in a signal-peptide dependent, but not sufficient, manner. This finding is surprising because RhCMV additionally expresses the HCMV US2-US11 homologs that also interfere with MHC-I stability and assembly. The VIHCE-encoding rh178 is so far unique to RhCMV suggesting that rh178 represents an adaptation to the evolutionary pressure of the non-human primate MHC system. Our previous observations [21] suggested that the immune evasion mechanisms encoded by the US2-US11 region predate the separation of human and old-world non-human primates which is assumed to have taken place about 25 million years ago [32]. Recent sequence analysis of the MHC-I locus in RM revealed that the MHC-I has undergone a tremendous change since then. Whereas a typical human or ape haplotype contains “only” six active MHC-I genes, as many as 22 different MHC-I genes are expressed in rhesus. Moreover, the sequence divergence was estimated to be 10-fold higher and genes have been duplicated at an approximately three times greater rate than in humans [33],[34]. Thus, it is conceivable that the additional MHC-I genes forced RhCMV to evolve additional countermeasures. It is known that polymorphic MHC-I proteins are differentially affected by US2 and US11 of HCMV [35],[36], although the exact rules of this discrimination still need to be determined. Moreover, each of the US6-family viral immune modulators interferes at a distinct step during the assembly cascade [37]. Allele-specificity has also been reported for MCMV which contains three genes [38], unrelated to either the US6-family or VIHCE, and each of three MCMV-gene products interferes with a different step of MHC-I assembly [39]. Thus, it seems that CMVs optimize their interference mechanisms, both within a given organism by sequentially attacking MHC molecules during assembly and within a given population by broadening the allele-specificities of these attacks. This conclusion is also supported by our finding that RhCMV lacking either rh178 or RhUS2-11 only partially suppressed MHC-I assembly and transport compared to WT RhCMV. This is either due to differences in allele-specificity within a given animal or an incomplete elimination of all alleles. The finding that RhCMV has a larger number of gene products interfering with MHC-I assembly than either HCMV or MCMV thus correlates with the observation that RM have a larger number of active MHC-I alleles than either human or mouse.

The extracellular domains of MHC-I, particularly the peptide-binding regions, are highly polymorphic and evolve rapidly. In contrast, the cleaved signal peptide is highly conserved among different MHC-I alleles including the RM MaMu and the human HLA genes [33]. Many signal peptides for MaMu-I, MaMu-3 and MaMu-A show less than 3 amino-acids difference to either HLA-A, B or C alleles and some MaMu-SPs are identical to HLA-SPs [40]. A possible reason for the high conservation of HLA signal peptide sequences is the fact that a conserved nona-peptide (VMAPRTLLL in the HLA-A3 sequence) is presented by the non-polymorphic HLA-E molecule to the negative signaling receptor CD94/NKG2A or C of NK cells [41]. This system seems to be conserved in RM, although some alleles start at the methionine within the peptide [33]. Interestingly, the SP of the HCMV UL40 glycoprotein contains this nona-peptide which is presented by HLA-E in HCMV-infected cells in a TAP-independent fashion [42],[43]. By loading the decoy peptide onto HLA-E, HCMV is thought to prevent the “missing self” stimulation of NK cells by MHC-I downregulation. Importantly, this nona-peptide is also encoded within the SP of Rh67 of RhCMV which otherwise shares only 19% identity with UL40 [12]. Since VIHCE requires polypeptide sequence beyond the SP in MHC-I HCs, the Rh67 protein is likely resistant to VIHCE despite containing a similar SP sequence.

The MHC-I SP mimic contained in UL40 sets precedence for CMV taking advantage of the highly conserved SP to escape the cellular immune response. Different from UL40 however, rh178 does not mimic the SP, but seems to rely at least in part on this conserved sequence to broadly eliminate HCs. VIHCE is clearly different from any other previously described immune modulatory mechanism since the ER-localized protein rh178 interferes with HC expression after the onset but prior to the completion of translation. One possible mechanism is that rh178 inhibits translation at a step that occurs after the SRP targets the nascent polypeptide/ribosomal complex to the ER membrane-localized SRP receptor. During this process, translation is arrested until SRP is released upon GTP hydrolysis and SEC61 binding [31],[44]. A possible scenario is that rh178 interacts with the SRP/nascent polypeptide/ribosome complex at the ER-membrane thus prolonging translational arrest. Alternatively, rh178 could prevent this complex interaction with the SEC61 translocon in ER-membrane. Conceivably, rh178 could also interfere with the translocation of HC in a manner similar to cotransin, a small molecule translocation inhibitor, which specifically interferes with binding of certain SPs to a SEC61 subunit [45]. The ensuing translocational stalling results in co-translational degradation by the proteasome, a process that involves cytosolic chaperones [46]. For non-stop RNA it was recently also shown that translational arrest results in protein fragments that are rapidly degraded by the proteasome [47]. Therefore, it seems likely that HC translation intermediates are degraded by the proteasome despite the fact that we were unable to detect a degradation intermediate in the presence of proteasome inhibitors. Possible reasons why such breakdown products were not identified are their potentially small and heterogenous size and their extremely rapid degradation. HC-derived intermediates might also lack the epitopes recognized by the HC-specific antibodies used in this study.

Targeted disruption of protein translation by a viral protein has so far not been described as an immune evasion strategy. However, it was recently shown that the microRNA miR-UL112 of HCMV inhibits the translation of MHC-I-related chain B (MICB), a ligand for the activating NK cell receptor NKG2D [48]. Thus, CMVs seem to interfere at multiple levels and by multiple strategies with translation of immune stimulatory genes. The virus might thereby employ or mimic cellular pathways of translational or translocational regulation. Further elucidation of the molecular events of VIHCE might thus reveal previously unrecognized host cell mechanisms of translational and translocational control.

## Materials and Methods

### Cells and viruses

Telomerized rhesus fibroblasts (TRFs) [49] and telomerized human fibroblasts (THFs) were obtained from Jay Nelson and maintained in Dulbecco's modified eagle's medium (DMEM) with 10% fetal bovine serum, 100U/mL penicillin and 100ug/mL streptomycin. RhCMV strain 68.1 was obtained from Scott Wong [12] and propagated in TRFs. Recombinant RhCMVs were created as described in the supplemental methods using the RhCMV BAC obtained from Peter Barry [23]. Recombinant rh178 adenoviruses were created using the AdEasy vector system according to the manufacturers protocol (Stratagene). Adenoviruses AdTrans and AdUS11 were obtained from David Johnson.

### Plasmids and Nucleofection

HLA-A3 and CD4 constructs were expressed from a modified version of pCDNA3.1(-) (Invitrogen, Carlsbad, CA) in which the CMV promoter was replaced with the EF1α promoter (obtained from Jay Nelson) to create pEF1α. HLA-A3 was obtained by PCR from Jurkat T-cell cDNA using the forward primer 5′ctggaattcatggccgtcatggcgccccgaac and the reverse primer 5′gtcggatcctcacactttacaagctgtgag to amplify the coding region only or the reverse primer 5′gtcggatccttaggaatcttctcc to include the 3′UTR. pEF1α expression plasmids were electroporated into TRFs using the AMAXA Nucleofector II (AMAXA Biosystems, Gaithersburg, MD) using cell line solution L and the T-030 program. 1e6-2e6 TRFs were resuspended in 100 µl AMAXA solution and 2 µg expression plasmid. After electroporation cells were recovered in 500 µl RPMI for 45min at 37°C, and then plated in prewarmed complete DMEM. Transfection efficiency was monitored with a GFP reporter and was consistently >90%. Infections with RhCMV were performed 24 hours after electroporation.

### Metabolic labeling and immunoprecipitation

Cells were starved for 30-min, except where noted, using DMEM without serum, methionine (Met) or cysteine (Cys). Labeling was performed for indicated times using Pro-mix ^35^S-Met/Cys (GE Healthcare) at 400 µCi/mL. To chase the label, cells were washed 3× in phosphate buffered saline (PBS) followed by incubation at 37°C in DMEM with 10% FBS containing 90 µg/mL Met and 188 µg/mL Cys. For NP-40 lysis, cells were lysed for 30 minutes at 4°C in 1% NP-40 in PBS with complete protease-inhibitor cocktail (Roche). For SDS lysis, cells were lysed for 10 minutes at 25°C in 0.6% SDS in PBS with complete protease-inhibitor cocktail, then diluted in 3× volume of 1.2% triton X-100 in PBS prior to immunoprecipitation. For glycosidase treatment, PNGase was obtained from NEB and used according to the manufacturers protocol after NP-40 lysis.

### Antibodies

Polyclonal sera K455 recognizes both chains of the MHC-I heterodimer, assembled and unassembled (obtained from Per Peterson) [24]. HC-10 only recognizes free MHC-I heavy chains [25]. HLA-A3 antibody was purified from the GAP A3 hybridoma, obtained from ATCC (HB-122). Antibodies to Calreticulin, Transferrin, Vimentin, HA and FLAG were obtained, respectively, from Stressgen (Victoria, BC), Zymed (S. San Francisco, CA), Biomeda (Burlingame, CA), Santa Cruz, and Sigma. Human CD4 antibody (AHS0412) was obtained from Invitrogen. Secondary Alexa Fluor-conjugated antibodies 594 goat anti-rabbit and 488 goat anti-mouse were obtained from Invitrogen.

### Polyribosome fractionation and northern blots

Approximately 5×10^6^ TRFs were either Mock-infected or RhCMV-infected for 24 hours. Fresh media was placed on the cells for 45-minutes, and cells were placed on ice and washed 2× with cold PBS containing 0.1 mg/ml cycloheximide (Sigma). All subsequent steps were performed at 4°C. Cells were lysed for 10 min using 600 µl of polysome lysis buffer (15mM Tris, pH 7.4, 15mM MgCl_2_, 0.3M NaCl, 1% Triton x-100, 0.1 mg/mL cycloheximide, 1 mg/mL heparin). Lysates were cleared at 12,000× g for 10 min. The supernatant was layered onto the top of a 10–50% sucrose gradient composed of sucrose in polysome lysis buffer excluding Triton x-100. The gradients were centrifuged at 35,000 rpm in a Sorvall SW-41 rotor for 3 hours. 750 µl fractions were collected from the top of the gradient. After adding 4.25ml of 5.65M guanidine HCl, each fraction was ethanol precipitated (−20°C overnight). RNA was pelleted at 15,000× g for 30 min, washed with 70% ethanol, dried at 25°C, and resuspended in 400 µl RNAse-free water. RNA was then re-precipitated by adding 40 µl 0.3M sodium acetate and 900 µl 100% ethanol, washed with 70% ethanol and resuspended in 50 µl RNAse-free water.

For Northern blotting, 10 µl of each fraction was separated on a denaturing 1% agarose gel containing 1× MESA (Boston BioProducts, Worcester, MA) and 3.7% formaldehyde and transferred to Immobilon-Ny+ nylon membrane (Millipore) by capillary blotting in 20×SSC. RNA was fixed by air drying at 25°C for 30 min and baking at 80°C for 2 hours. Radiolabeled probes were generated by random priming. After denaturing at 100°C for 10 min, the probe was chilled on ice and added to 5mL ExpressHyb hybridization solution (Clontech) for hybridization. Membranes were pre-hybridized for 30 min at 68°C followed by probe hybridization for 2 hours, rinsed and washed twice with 2× SSC, 0.05%SDS followed by two washes in 0.1× SSC, 0.1% SDS.

### Immunofluorescence

Transfected cells were fixed with 3.7% formaldehyde for 40 minutes, washed twice with PBS, quenched with 50mM NH_4_Cl for 10 min, washed twice with PBS, and permeabilized with 0.1% Triton X-100 in PBS for 7 min prior to staining.

### RACE

Total RNA from TRFs infected with WT RhCMV (or RhCMV lacking *rh175–178* as a negative control) for 24 hours was used. For 3′ RACE, cDNA was synthesized using an oligo-dT anchor (5′gaccggatccgaattcgtcgacttttttttttttttttv). PCR was performed from cDNA using a PCR anchor primer (5′-gaccggatccgaattcgtcgac) and a gene specific primer. For 5′ RACE, cDNA was synthesized with a gene specific primer (*rh178* 5′-catttgcatgcagctgtgcg). 10 µg cDNA was then treated with terminal deoxynucleotidyl transferase and 0.5mM dATP at 37°C for 30 min, followed by purification and PCR using a nested gene specific primer (*rh178* 5′-gcgcgaaacacgcgtttgc) and the oligo-dT anchor.

## Supporting Information

Figure S1 HC synthesis is not delayed nor rapidly degraded upon synthesis during RhCMV infection. A) HC synthesis is not delayed. Cells were radiolabeled for 10 min followed by chase of indicated times. After SDS lysis, IP was performed using HC-10 antibody, which recognizes free MHC-I HC. (*) A non-MHC-I-specific band indicating protein loading. B) HC is not rapidly degraded upon synthesis. TRFs were infected with the indicated virus, radiolabeled for 1 min, chased for 30 min, lysed with NP-40 lysis buffer and IP performed with K455.
(1.59 MB TIF)

Figure S2 RhCMV contains viral antibody binding proteins that are not specific to the immunoprecipitated antigen. Complete autoradiograph from Fig 2B showing pulse-chase and IP during infection with RhCMV Δ158–180 and Δ158–180, ΔRhUS2-11. Indicated on the left side are molecular weight estimates. This indicates the viral antibody binding proteins that are not shown in IPs from other figures since they are non-specific to the immunoprecipitated antigen.
(1.97 MB TIF)

Figure S3 *rh178* is expressed as an early gene transcript. Northern blot analysis of rh178 and Rh156 (IE1) at 4 and 24 hours post infection. Cyclohexamide (CHX) and phosphonoacetic acid (PAA) were included where indicated. Note that PAA did not inhibit VIHCE expression indicating that VIHCE is not a late gene. In contrast, CHX inhibited VIHCE expression indicating that VIHCE is not an immediate early gene.
(0.96 MB TIF)

Protocol S1 Supplemental materials and methods and figure legends.
(0.04 MB DOC)

Table S1 Sequences of the recombination portion of the BAC mutagenesis primers.
(0.03 MB DOC)
