# Supplementary figures and images for: Signal Peptide-Dependent Inhibition of MHC Class I Heavy Chain Translation by Rhesus Cytomegalovirus
Source: PLoS Pathog. 2008 Oct 3;4(10):e1000150. doi: 10.1371/journal.ppat.1000150 (PMC2542416; doi:10.1371/journal.ppat.1000150)

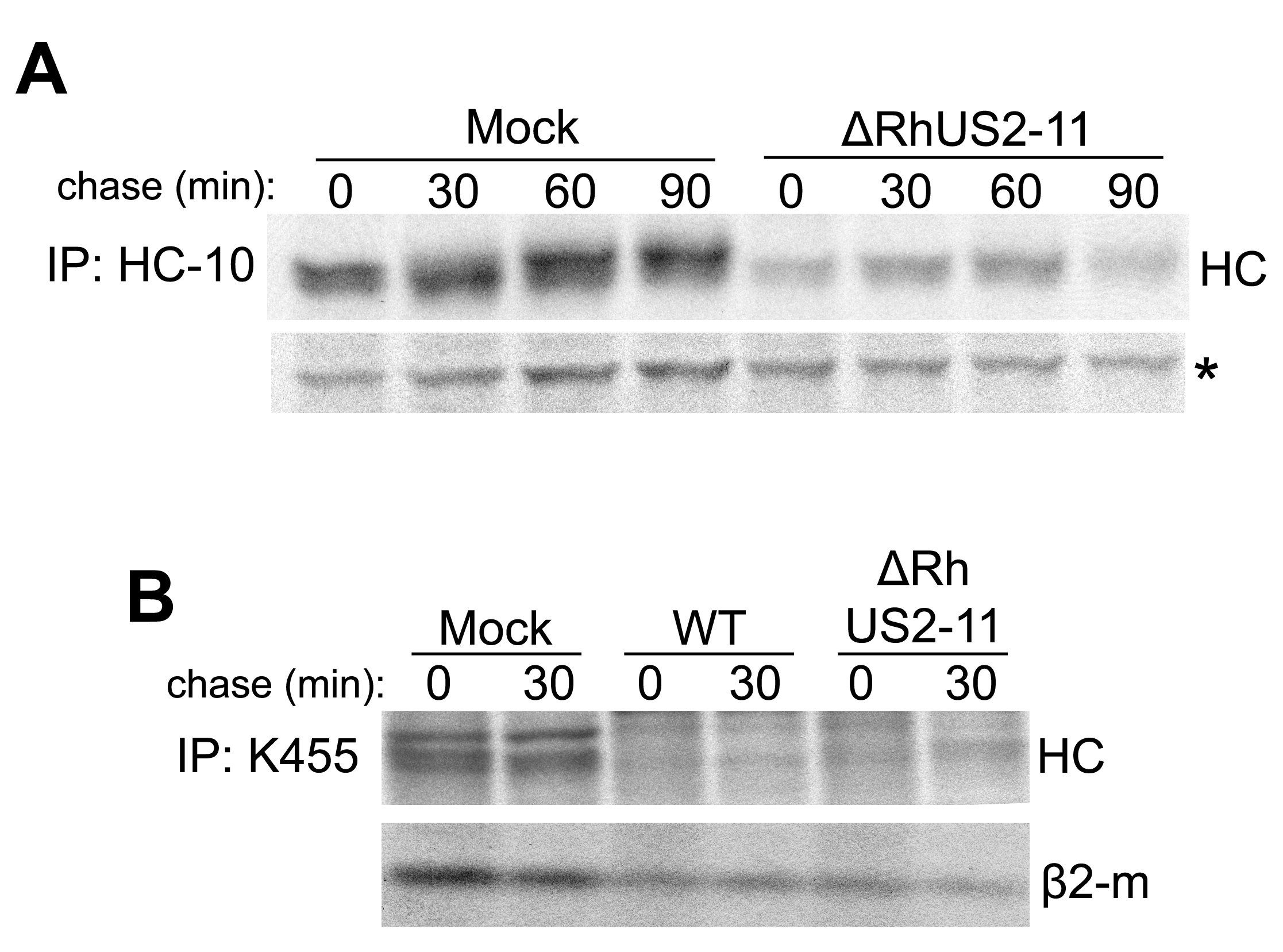

Supplement: Figure S1 — HC synthesis is not delayed nor rapidly degraded upon synthesis during RhCMV infection. A) HC synthesis is not delayed. Cells were radiolabeled for 10 min followed by chase of indicated times. After SDS lysis, IP was performed using HC-10 antibody, which recognizes free MHC-I HC. (*) A non-MHC-I-specific band indicating protein loading. B) HC is not rapidly degraded upon synthesis. TRFs were infected with the indicated virus, radiolabeled for 1 min, chased for 30 min, lysed with NP-40 lysis buffer and IP performed with K455. (1.59 MB TIF) [file ppat.1000150.s001.tif]

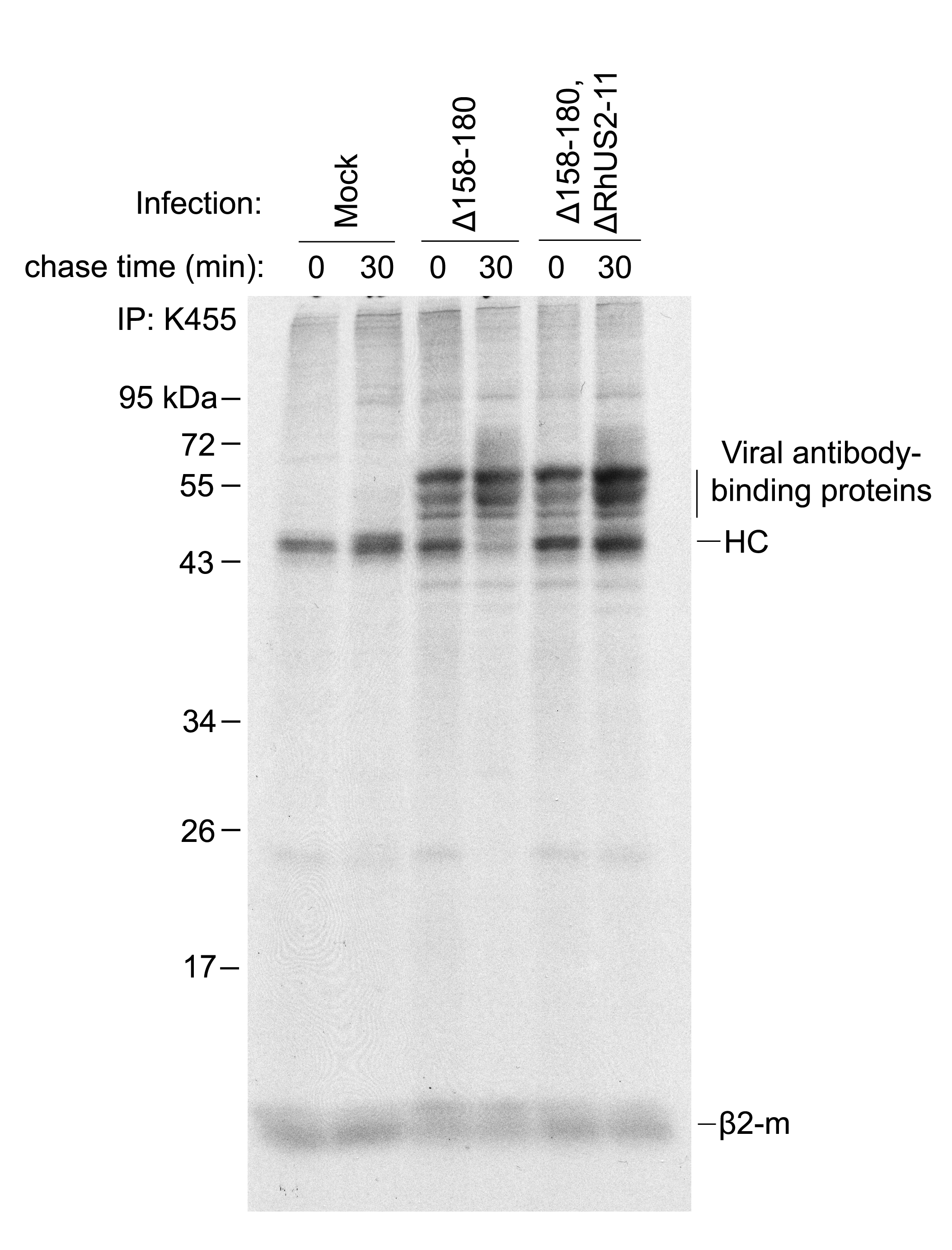

Supplement: Figure S2 — RhCMV contains viral antibody binding proteins that are not specific to the immunoprecipitated antigen. Complete autoradiograph from Fig 2B showing pulse-chase and IP during infection with RhCMV Δ158–180 and Δ158–180, ΔRhUS2-11. Indicated on the left side are molecular weight estimates. This indicates the viral antibody binding proteins that are not shown in IPs from other figures since they are non-specific to the immunoprecipitated antigen. (1.97 MB TIF) [file ppat.1000150.s002.tif]

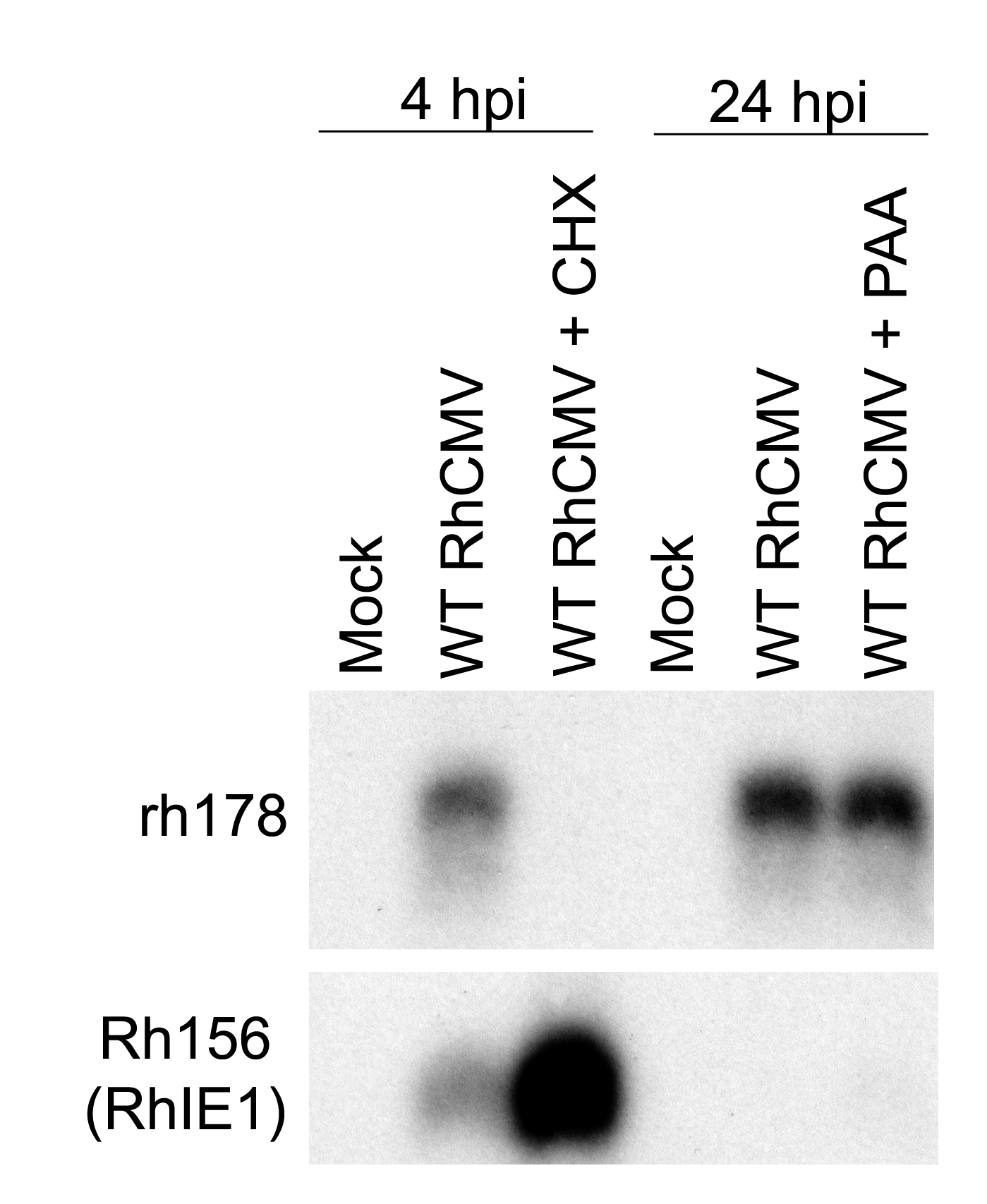

Supplement: Figure S3 — rh178 is expressed as an early gene transcript. Northern blot analysis of rh178 and Rh156 (IE1) at 4 and 24 hours post infection. Cyclohexamide (CHX) and phosphonoacetic acid (PAA) were included where indicated. Note that PAA did not inhibit VIHCE expression indicating that VIHCE is not a late gene. In contrast, CHX inhibited VIHCE expression indicating that VIHCE is not an immediate early gene. (0.96 MB TIF) [file ppat.1000150.s003.tif]
