# Supplementary material for: Signal Peptide-Dependent Inhibition of MHC Class I Heavy Chain Translation by Rhesus Cytomegalovirus
Source: PLoS Pathog. 2008 Oct 3;4(10):e1000150. doi: 10.1371/journal.ppat.1000150 (PMC2542416; doi:10.1371/journal.ppat.1000150)
Supplement: Protocol S1 — Supplemental materials and methods and figure legends. (0.04 MB DOC) [file ppat.1000150.s004.doc]

**Supplemental Materials and Methods**

**Recombinant virus**

RhCMV BAC was maintained in EL250 bacterial cells which contain heat-inducible λ-recombination (rec) genes and an arabinose-inducible FLP recombinase (Lee et al., 2001). All bacterial growth was performed at 30ºC unless otherwise noted. To induce the rec genes, a 200ml culture of EL250s was grown in LB to OD=0.35 at 600nm and placed at 42ºC for 17 min. Bacteria were then placed on ice for 10 min and made electrocompetent by washing 4x with 250ml 10% glycerol and snap freezing in liquid nitrogen.

To make the PCR product for recombination, primers containing 40-50bp of homology to the sequences flanking the RhCMV region to be removed were used to amplify a Kanamycin (Kan) resistance cassette from plasmid pCP015 (Cherepanov and Wackernagel, 1995). The pCP015 forward primer binding site (5’gtaaaacgacggccagt) and reverse primer binding site (5’gaaacagctatgaccatg) were added to the 3’ end of the mutagenesis primers.

Competent EL250s containing WT RhCMV BAC were then electroporated with the PCR product for recombination using a MicroPulser (Biorad, Hercules, CA) and selected for Kan and Chloramphenicol (Cm) resistance at 30ºC on LB agar. Clones were first screened to insure no plasmid contamination. To induce the FLP recombinase to excise the Kan^r^ cassette, clones were grown in LB with Cm only to OD=0.5 at 600nm and incubated with 1mg/mL arabinose for 1 hr, diluted 1:10 in LB with Cm, and plated on LB agar with Cm. Following replica plating on LB agar with Kan and Cm, selected colonies that had lost Kan^r^ were characterized by restriction digest, southern blot, and partial sequencing. Virus was reconstituted by electroporation of TRFs with 5-10μg of BAC DNA. The Kan^r^ cassette was not removed from the deletion viruses shown in Fig 3A in order to reduce the total amount of DNA removed from the genome. Sequences for the primers used for all recombinant BACs can be found in Supplemental Table 1.

For the double deletion virus Δ158-180,ΔRhUS2-11, we used the ΔRhUS2-11 BAC as a parent and deleted Rh158-180 as detailed above. We also did not remove the Kan resistance cassette in this BAC due to the presence of the FRT recombination site from the RhUS2-11 deletion and to lessen the amount of total DNA removed from the genome.

**Metabolic labeling for 1-min**

For 1-minute pulses, cells were trypsinized, pelleted, and resuspended in 200μl DMEM (- Met/Cys). 500μCi/mL ^35^S-Met/Cys was added and the cells placed at 37ºC. After 1-min, 1ml of chase media was added to quench, cells were pelleted, washed 2x in chase media, lysed or resuspended in 1ml chase medium for the indicated time.

**Supplemental References**

Cherepanov, P. P., and Wackernagel, W. (1995). Gene disruption in Escherichia coli: TcR and KmR cassettes with the option of Flp-catalyzed excision of the antibiotic-resistance determinant. Gene *158*, 9-14.

Lee, E. C., Yu, D., Martinez de Velasco, J., Tessarollo, L., Swing, D. A., Court, D. L., Jenkins, N. A., and Copeland, N. G. (2001). A highly efficient Escherichia coli-based chromosome engineering system adapted for recombinogenic targeting and subcloning of BAC DNA. Genomics *73*, 56-65.

**Supplemental Figure Legends**

**Supplemental Figure 1. HC synthesis is not delayed nor rapidly degraded upon synthesis.**

A) HC synthesis is not delayed. Cells were radiolabeled for 10 min followed by chase of indicated times. After SDS lysis, IP was performed using HC-10 antibody, which recognizes free MHC-I HC. (*) A non-MHC-I-specific band indicating protein loading. B) HC is not rapidly degraded upon synthesis. TRFs were infected with the indicated virus, radiolabeled for 1 min, chased for 30 min, lysed with NP-40 lysis buffer and IP performed with K455.

**Supplemental Figure 2. RhCMV contains viral antibody binding proteins that are not specific to the immunoprecipitated antigen.**

Complete autoradiograph from Fig 2B showing pulse-chase and IP during infection with RhCMV Δ158-180 and Δ158-180, ΔRhUS2-11. Indicated on the left side are molecular weight estimates. This indicates the viral antibody binding proteins that are not shown in IPs from other figures since they are non-specific to the immunoprecipitated antigen.

**Supplemental Figure 3. *rh178* is expressed as an early gene transcript.**

Northern blot analysis of rh178 and Rh156 (IE1) at 4 and 24 hours post infection. Cyclohexamide (CHX) and phosphonoacetic acid (PAA) were included where indicated. Note that PAA did not inhibit VIHCE expression indicating that VIHCE is not a late gene. In contrast, CHX inhibited VIHCE expression indicating that VIHCE is not an immediate early gene.
