# Supplementary material for: Signal Peptide-Dependent Inhibition of MHC Class I Heavy Chain Translation by Rhesus Cytomegalovirus
Source: PLoS Pathog. 2008 Oct 3;4(10):e1000150. doi: 10.1371/journal.ppat.1000150 (PMC2542416; doi:10.1371/journal.ppat.1000150)
Supplement: Table S1 — Sequences of the recombination portion of the BAC mutagenesis primers. (0.03 MB DOC) [file ppat.1000150.s005.doc]

Supplemental Table 1. Sequences of the recombination portion of the BAC mutagenesis primers

| **Forward mutagenesis primer (5’-3’)** | **Reverse mutagenesis primer (5’-3’)** | **ORFs deleted** |
| --- | --- | --- |
| agaaatcgcaaagacattctggtttctgttttgtttcattt | aagcccttcacatgaatcccataaatcataaatgacaggttac | RhUS2-US11 |
| gcaagattacagagtgtaaaagacggcatgttatctacggta | cagggtacgagccgaattaacaggatagtcataggtaggt | Rh158-180 |
| gcaagattacagagtgtaaaagacggcatgttatctacggta | tctgctcacgagctgccctgaagacaactaaccaccacaacccgcgaat | Rh158-168 |
| ccgacggtgttttttatctgccattcttgctcgcgtcagtccatgcg | cagggtacgagccgaattaacaggatagtcataggtaggt | Rh167-180 |
| ccgacggtgttttttatctgccattcttgctcgcgtcagtccatgcg | ttattttaaattatgggctgggtacttacttgaaggtcaagagcacggcc | Rh167-174 |
| gtggtggctgctggccaagtaacgatccacaccatgaattgtcgcactag | cagggtacgagccgaattaacaggatagtcataggtaggt | Rh175-180 |
| gtggtggctgctggccaagtaacgatccacaccatgaattgtcgcactag | cgctccctcggcctgactgatgactagtcatcgcacgcctcttcccgcccgt | Rh175-178 |
| gggcgggaagaggcgtgcgatgactagtcatcagtcaggccgagggagcg | cagggtacgagccgaattaacaggatagtcataggtaggt | Rh179-180 |
| gtggtggctgctggccaagtaacgatccacaccatgaattgtcgcactag | cacattcatccgacacttttatacgaacaaactcgccagt | Rh175-177 |
| ggccatgaaaagtcctaggagaaacacaacgcaaagagca | tctgccgtatcggacaaccctacgccaagcgcacagctgc | Rh176-178 |
| ggtgatggcatgcgcttctgggtgaaagcgagcgtgtgcg | cgctccctcggcctgactgatgactagtcatcgcacgcctcttcccgcccgt | Rh177-178 |

Note: These sequences are only the primer portion containing homology to RhCMV and do not contain the pCP015 primer binding sites.
